# Revisiting a century of dermatology: an analysis of the themes in the articles of Anais Brasileiros de Dermatologia (1925–2025)^؆^

**DOI:** 10.1016/j.abd.2025.501197

**Published:** 2025-08-20

**Authors:** Thays Herbst Carvalho, Aluísio José de Oliveira Monteiro Neto, Laís Acioli Lins, Milena Aguiar Alencar de Oliveira, Mateus Harmad Char, Hélio Amante Miot

**Affiliations:** Department of Infectology, Dermatology, Imaging Diagnosis and Radiotherapy, Faculty of Medicina, Universidade Estadual Paulista, Botucatu, SP, Brazil

Dear Editor,

*Anais Brasileiros de Dermatologia* (ABD) constitute the main scientific journal of the Brazilian Society of Dermatology (SBD, *Sociedade Brasileira de Dermatologia*). Published since 1925, they play a fundamental role in disseminating knowledge in the field, contributing to professionals’ training and updating, in addition to providing a platform for the dissemination of original research, reviews, and relevant clinical cases.^1^

The ABD is indexed in the main international databases, making it the most influential dermatology journal in Latin America, and the only one indexed in the ISI-Web of Science database, which calculates journal impact factors.^2^

Over a century of its publication, the ABD has accumulated more than six thousand articles authored by more than ten thousand researchers. These studies have tracked changes in clinical practice, the emergence of new diseases, and technological innovations, consolidating the journal as an essential reference for dermatologists and researchers.^3^ At the same time, the ABD underwent adjustments to its editorial policy to adapt to the demands of a modern, dynamic journal with international reach and high scientific rigor.^4^, ^5^

Analysis of the titles of articles published in the ABD over time allows us to understand changes in dermatological practice and reflect epidemiological and social trends, as well as scientific advances of each period. This study aimed to conduct qualitative bibliometric research on the topics addressed in articles published by the ABD between 1925 and 2025.

The titles of articles published in the ABD between 1925 and April 1, 2025, were extracted from the website http://www.anaisdedermatologia.com.br/edicoes-anteriores. The articles were grouped into four periods: 1925–1950, 1951–1975, 1976–2000, and 2001–2025. The titles were normalized for grammatical uniqueness, and spelling variations (such as "syphilis" and "sífilis") and related terms (such as "leprosy," "*hanseníase*," and "*hansênico*") were manually identified. Generic words (such as "patient," "dermatosis," and "treatment"), prepositions, articles, conjunctions, and adverbs were excluded using ChatGPT 4.0®, which was also used to translate the titles available exclusively in English. The term groups were processed by WordArt (http://www.wordart.com/create) to generate word clouds with the most frequent terms (up to 100), which were identified in more than 1% of the titles.^6^

The frequencies of the 20 most frequently identified diseases in the titles were compared across the four periods using residual analysis corrected by the Holm-Bonferroni procedure.^7^ Significance was defined as p < 0.05.

The distribution of the 150 most frequent themes in each period is represented in the word clouds in Fig. 1. In the first period (1925–1950), infectious diseases such as treponematoses (syphilis, yaws, and pinta), lymphogranuloma venereum, leprosy, paracoccidioidomycosis, and leishmaniasis stood out. Arsenotherapy was used to treat treponematoses and leishmaniasis. Pemphigus foliaceus was the most important inflammatory disease.

**Fig. 1:**
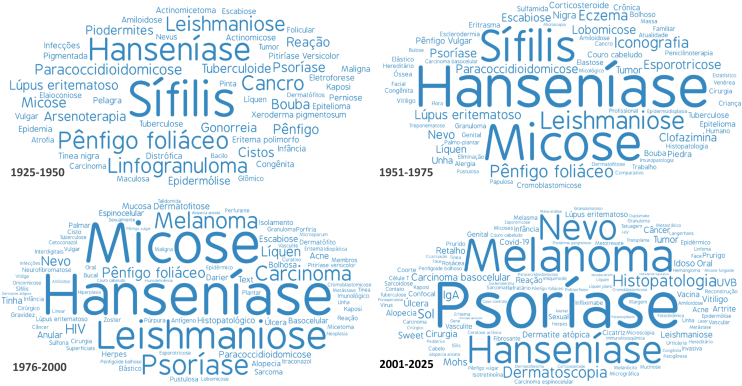
Word clouds representing the most frequently used terms in the titles of articles published in the *Anais Brasileiros de Dermatologia* in the four periods: 1925‒1950, 1951‒1975, 1976‒2000, 2000‒2025. The size of the words is proportional to their frequency of occurrence.

In the second period, infectious diseases continued to predominate, including leprosy, syphilis, leishmaniasis, paracoccidioidomycosis, chromomycosis, sporotrichosis, lobomycosis, and superficial mycoses. The introduction of penicillin changed the treatment of treponematoses, while corticosteroids and sulfones began to be used for inflammatory diseases and leprosy. Diseases such as eczema and psoriasis became more commonly cited.

In the third period, infectious diseases such as leprosy, leishmaniasis, and paracoccidioidomycosis still predominated. Psoriasis gained prominence, as did melanoma, carcinomas, and scabies. The HIV epidemic at the end of the last century stands out.

In the last period, psoriasis and neoplasms gained prominence over infectious diseases. Articles appeared on dermatoscopy, micrographic surgery, alopecia areata, and immunobiologicals. Articles involving COVID-19 in the last five years stand out.

Table 1 depicts the frequencies of the 20 diseases most frequently mentioned in article titles over time, highlighting the significant increase in the number of publications in the last period and the variation in the frequency of references to these diseases over time.

**Table 1:** Frequency of the 20 most commonly identified groups of dermatoses among the titles of articles in the *Anais Brasileiros de Dermatologia* (1925‒2025).

Disease	1925 to 1950 (n = 250)	1951 to 1975 (n = 468)	1975 to 2000 (n = 1.444)	2001 to 2025^a^ (n = 3.867)
Acne	1 (0.4%)	2 (0.4%)	18 (1.2%)	60 (1.6%)
Alopecia areata	0 (-)	0 (-)	6 (0.4%)	32 (0.8%)
Atopic dermatitis	0 (-)	3 (0.6%)	7 (0.5%)	61 (1.6%)
Basal cell carcinoma	0 (-)	3 (0.6%)	15 (1.0%)	81 (2.1%)
Scabies	2 (0.8%)	7 (1.5%)	18 (1.2%)	11 (0.3%)
Sporotrichosis	1 (0.4%)	9 (1.9%)	11 (0.8%)	27 (0.7%)
Leprosy	21 (8.4%)	28 (6.0%)	64 (4.4%)	125 (3.2%)
Herpes simplex	0 (-)	0 (-)	6 (0.4%)	11 (0.3%)
Leishmaniasis	8 (3.2%)	15 (3.2%)	62 (4.3%)	75 (1.9%)
Lupus erythematosus	2 (0.8%)	6 (1.3%)	14 (1.0%)	42 (1.1%)
Melanoma	0 (-)	1 (0.2%)	25 (1.7%)	160 (4.1%)
Superficial Mycosis	5 (2.0%)	24 (5.1%)	63 (4.4%)	47 (1.2%)
Paracoccidioidomycosis	9 (3.6%)	8 (1.7%)	28 (1.9%)	25 (0.6%)
Bullous pemphigoid	0 (-)	1 (0.2%)	6 (0.4%)	28 (0.7%)
Pemphigus foliaceus	8 (3.2%)	12 (2.6%)	22 (1.5%)	23 (0.6%)
Psoriasis	5 (2.0%)	8 (1.7%)	38 (2.6%)	197 (5.1%)
Syphilis	46 (18.4%)	14 (3.0%)	12 (0.8%)	26 (0.7%)
Urticaria	0 (-)	1 (0.2%)	3 (0.2%)	42 (1.1%)
Vitiligo	1 (0.4%)	3 (0.6%)	9 (0.6%)	71 (1.8%)
Zoster	0 (-)	2 (0.4%)	12 (0.8%)	5 (0.1%)

a Data up to 04/01/2025; Cells in GREEN: lowest frequency (p < 0.05); Cells in RED: highest frequency (p < 0.05).

In the last century, Brazil underwent an epidemiological transition marked by a reduction in infectious diseases as well as an increase in chronic non-communicable diseases and neoplasms, driven by population aging and lifestyle changes. This process occurred in parallel with social and cultural transformations, such as accelerated urbanization, improved sanitation, and advances in medical science.^8^

Penicillin arrived in Brazil in the 1950s, which may explain the reduction in publications on treponematoses from the third period onward. Similarly, multidrug therapy for leprosy was instituted in 1986, drastically reducing its morbidity and prevalence, reflecting lower numbers of publications in the last period.

Immunobiologicals, which revolutionized psoriasis, have been available in Brazil since the first decade of the millennium, which may explain the recent increase in publications on the disease. The same can be said for modern treatments for atopic dermatitis, urticaria, vitiligo, and alopecia areata.

The progressively greater longevity of the population and the development of Brazilian dermatological surgery over the last 50 years may be reasons for the progressive increase in the frequency of publications in oncology and bullous pemphigoid.

Between the 1960s and 1980s, cases of sporotrichosis emerged in the Southeast region, mainly among farmers and vegetable handlers, sparking the interest of dermatologists. The increasing urbanization of the Brazilian population, associated with the availability of effective outpatient treatments, may explain the recent reduction in publications on paracoccidioidomycosis, pemphigus foliaceus, and leishmaniasis.

The reduced prominence of infectious diseases, despite the greater focus on inflammatory dermatoses and neoplasms, reflects not only changes in disease epidemiology but also research funding priorities.^6^, ^9^ Over time, the frequency of these topics in medical journals illustrates the dynamic nature of medicine, where public health demands and pharmaceutical advances drive scientific discourse.^10^

Finally, ABD publications have accompanied global health crises, such as syphilis, AIDS, and the COVID-19 pandemic. Similarly, they have reflected the evolution of therapies for venereal diseases, leishmaniasis, leprosy, psoriasis, atopic dermatitis, and alopecia areata.

This study has limitations related to the individual analysis of terms without assessing their semantic relationships, variations in medical terminology, and editorial rules over time. Future studies should be conducted based on the analysis of full texts, considering their impact, citations, and types of publication, for a more detailed understanding of the variations in editorial practices in Brazilian dermatology.

In conclusion, given the scenario of constant scientific evolution, understanding the trends in academic production allows not only revisiting the trajectory of Brazilian dermatology but also identifying knowledge gaps and directing future research to meet the emerging demands of the specialty.

## Financial support

CNPq (306358/2022-0) – Hélio Amante Miot is a CNPq research fellow.

## Authors’ contributions

Hélio Amante Miot: Design and planning of the study; analysis and interpretation of data; statistical analysis; drafting and editing of the manuscript; critical review of the literature; critical review of the manuscript; approval of the final version of the manuscript.

Thays Herbst Carvalho: Collection of data; analysis and interpretation of data; statistical analysis; drafting and editing of the manuscript; critical review of the literature; critical review of the manuscript; approval of the final version of the manuscript.

Aluísio José de Oliveira Monteiro Neto: Collection of data; analysis and interpretation of data; drafting and editing of the manuscript; critical review of the literature; critical review of the manuscript; approval of the final version of the manuscript.

Laís Acioli Lins: Collection of data; analysis and interpretation of data; drafting and editing of the manuscript; critical review of the literature; critical review of the manuscript; approval of the final version of the manuscript.

Milena Aguiar Alencar de Oliveira: Collection of data; analysis and interpretation of data; drafting and editing of the manuscript; critical review of the literature; critical review of the manuscript; approval of the final version of the manuscript.

Mateus Harmad Char: Collection of data; analysis and interpretation of data; drafting and editing of the manuscript; critical review of the literature; critical review of the manuscript; approval of the final version of the manuscript.

## Research data availability

The entire dataset supporting the results of this study was published in this article.

## Editor

Hiram Larangeira de Almeida Jr.

## Conflicts of interest

None declared.
